# Paratuberculosis in South American camelids: two independent cases in alpacas in Germany

**DOI:** 10.1186/s12917-024-04414-z

**Published:** 2024-12-04

**Authors:** Heike Köhler, Jana Müller, Elena Kloß, Petra Möbius, Stefanie A. Barth, Marlene Sickinger, Nicole Gies, Carsten Heydel, Martin Peters

**Affiliations:** 1 Institute of Molecular Pathogenesis, Friedrich-Loeffler-Institut - Federal Research Institute for Animal Health (FLI), Jena, Germany; 2 National Reference Laboratory for Paratuberculosis, Institute of Molecular Pathogenesis, Friedrich-Loeffler-Institut - Federal Research Institute for Animal Health (FLI), Jena, Germany; 3 National Reference Laboratory for Bovine Tuberculosis, Institute of Molecular Pathogenesis, Friedrich-Loeffler-Institut - Federal Research Institute for Animal Health (FLI), Jena, Germany; 4 Institute for Veterinary Pathology, Justus-Liebig-University of Giessen, Giessen, Germany; 5 Clinic for Ruminants, Justus-Liebig-University of Giessen, Giessen, Germany; 6 Tierpark Hamm, Hamm, Germany; 7 Institute for Hygiene and Infectious Diseases of Animals, Justus Liebig University Giessen, Giessen, Germany; 8 Chemical and Veterinary Investigation Office Westphalia, Arnsberg, Germany

**Keywords:** Paratuberculosis, Alpaca, SAC, Germany, Isolate genotyping, Pathology, Mycobacterium avium, Johne´s disease, MAP-C, SNP-based assay, MIRU-VNTR, SSR

## Abstract

**Background:**

Paratuberculosis, caused by *Mycobacterium avium* subspecies *paratuberculosis* (MAP), is a chronic granulomatous enteritis that affects domestic and wild ruminants and camelids. The disease has rarely been reported in alpacas in Germany. This publication describes epidemiologically independent cases of paratuberculosis in two alpacas in Germany.

**Case presentation:**

Two alpacas, a 26-year-old female zoo animal (case 1) and a 2.5-year-old breeding stallion from a private owner (case 2), presented with progressive emaciation, leading to death (case 2) or euthanasia (case 1) because of deteriorating general condition. In both cases typical granulomatous lesions in the intestinal mucosa and mesenteric lymph nodes were found. In case 2, other lymph nodes were severely enlarged and MAP was detected in the mandibular lymph node, lung, and liver by qPCR. The MAP isolates differed between the alpacas, with two distinct phylogenetic clades (Clade 1 and 8) within Subgroup A of the MAP-C type group and two distinct INMV profiles (INMV 2 and 1) found. These genotypes have been identified in cattle and goats in different regions in Germany. The genotype isolated from case 1 has been detected in goats from the zoo since 2011, indicating transmission between these species.

**Conclusions:**

MAP can cause severe clinical disease in alpacas of variable age and under different husbandry conditions. Therefore, paratuberculosis should be considered for differential diagnosis in alpacas with emaciation and poor general condition. Although not definitely shown, cross-species infection between ruminant species and camelids is exceedingly likely.

## Background

Paratuberculosis is a WOAH listed infectious disease [1] that affects domestic and wild ruminants, such as cattle, goats and sheep [2]. The disease is very common in livestock, in particular in countries with large ruminant populations, resulting in considerable economic losses for the farmers [2]. In Germany, significant regional differences in between-herd prevalence have been observed, ranging from 3.6 to 50.1% in cattle and from 20.6 to 65.0% in small ruminants [3–5]. The disease is caused by *Mycobacterium avium* subsp. *paratuberculosis* (MAP) and manifests itself as a chronic granulomatous enteritis and lymphadenitis, causing protein-losing enteropathy and severe weight loss [2]. After a prolonged, clinically inconspicuous course, rather non-specific symptoms appear, such as emaciation, subcutaneous oedema and watery diarrhoea, which is more common in cattle than in goats and sheep [2]. Paratuberculosis affects also South American Camelids (SAC) [6, 7], which often do not develop the intractable diarrhoea that accompanies the weight loss in cattle [8].

In recent years, SAC have been increasingly introduced into North America and Europe [9], including Germany [10]. They are classified into four different species: alpacas (*Vicugna pacos*), vicuñas (*Vicugna vicugna*), llamas (*Lama glama*) and guanacos (*Lama guanicoe*) [9]. The number of SAC in Germany is about 25,000, as estimated by a German breeding organization, the Alpaka Zucht Verband Deutschland [11]. They are kept in full- or part-time commercial farming for fiber production or for breeding purposes, for landscape management, for animal-assisted therapy, or simply as pets [10, 12, 13]. Although rarely in Germany, alpacas and llamas may also serve as meat producing animals [8].

Little is known about the distribution of MAP in SAC in their natural habitat, MAP has only been detected in a few studies in wild guanacos in Patagonia and Tierra del Fuego [6, 7, 14]. Similarly, there is limited data on the prevalence of paratuberculosis in German SAC. To date, to the best of our knowledge, there is only a single report of a clinical case of paratuberculosis in an zoo alpaca in Germany [15]. In a recent study involving 43 farms and zoos in Central Germany, MAP was not detected in composite faecal samples from fresh droppings from alpaca and llama animal groups [12]. This suggests that paratuberculosis is not widespread in these animals.

## Case presentation

### Case 1 – alpaca from a zoo

#### Anamnesis

The affected animal was a 26-year-old female alpaca originating from a zoo in North Rhine-Westphalia, where it was born. It was kept in an alpaca group consisting of seven adult alpacas (one male, six females) and two male crias (juvenile) born in 2022.

Alongside the alpacas the zoo kept, among other animals, a group of four female llamas and one female guanaco, a group of two female Bactrian camels, a group of Four-Horned goats and a petting zoo where Dwarf goats and Spotted Mountain sheep were socialized. All these animal groups were kept in separate open stables with separate outdoor enclosures, which were cleaned twice a day. The different enclosures had separate cleaning equipment and their own feed storage. The alpaca enclosure and the petting zoo are open to visitors.

The small ruminants were monitored annually for brucellosis, leukosis, Q-fever and paratuberculosis by individual serological tests. Over the years, individual cases of paratuberculosis had been diagnosed serologically only in goats (ID Screen Paratuberculosis Indirect, Innovative Diagnostics, France; Verification: IDEXX Paratuberculosis Verification, IDEXX, The Netherlands), which were subsequently removed. Until 2022, the alpacas were only tested for brucellosis and paratuberculosis as part of movement tests, until then with negative results. Annual monitoring was now introduced for them. In consultation with the regional veterinary office, the camelids were regularly vaccinated against Clostridial diseases using a polyvalent bacterin-toxoid vaccine (Covexin 10 or Covexin 8 depending on availability, Zoetis, Germany) and the ruminants of the petting zoo were vaccinated against Q-fever (Coxevac, Ceva, Germany).

In spring 2022, the female alpaca showed intermittent watery diarrhoea and emaciation over about six weeks and was finally euthanized on site by the zoo veterinarian due to deterioration of the health status. For euthanasia, the animal was first anaesthetised by intramuscular injection of 1 mg/kg body weight (bw) of ketamine (Anesketin, Dechra, Germany) and 0.05 mg/kg bw medetomidine (Sedator, Dechra, Germany). After loss of consciousness, it was euthanised by intravenous application of 3 ml/10 kg bw sodium phenobarbital (Euthadorm, CP-Pharma, Germany). During the clinical course, several faecal samples were submitted for parasitological and microbiological examination to clarify the causes of diarrhoea, but without any significant findings. Other laboratory data is not available. The carcass was submitted to the regional veterinary diagnostic laboratory for necropsy.

#### Pathology

**Fig. 1:**
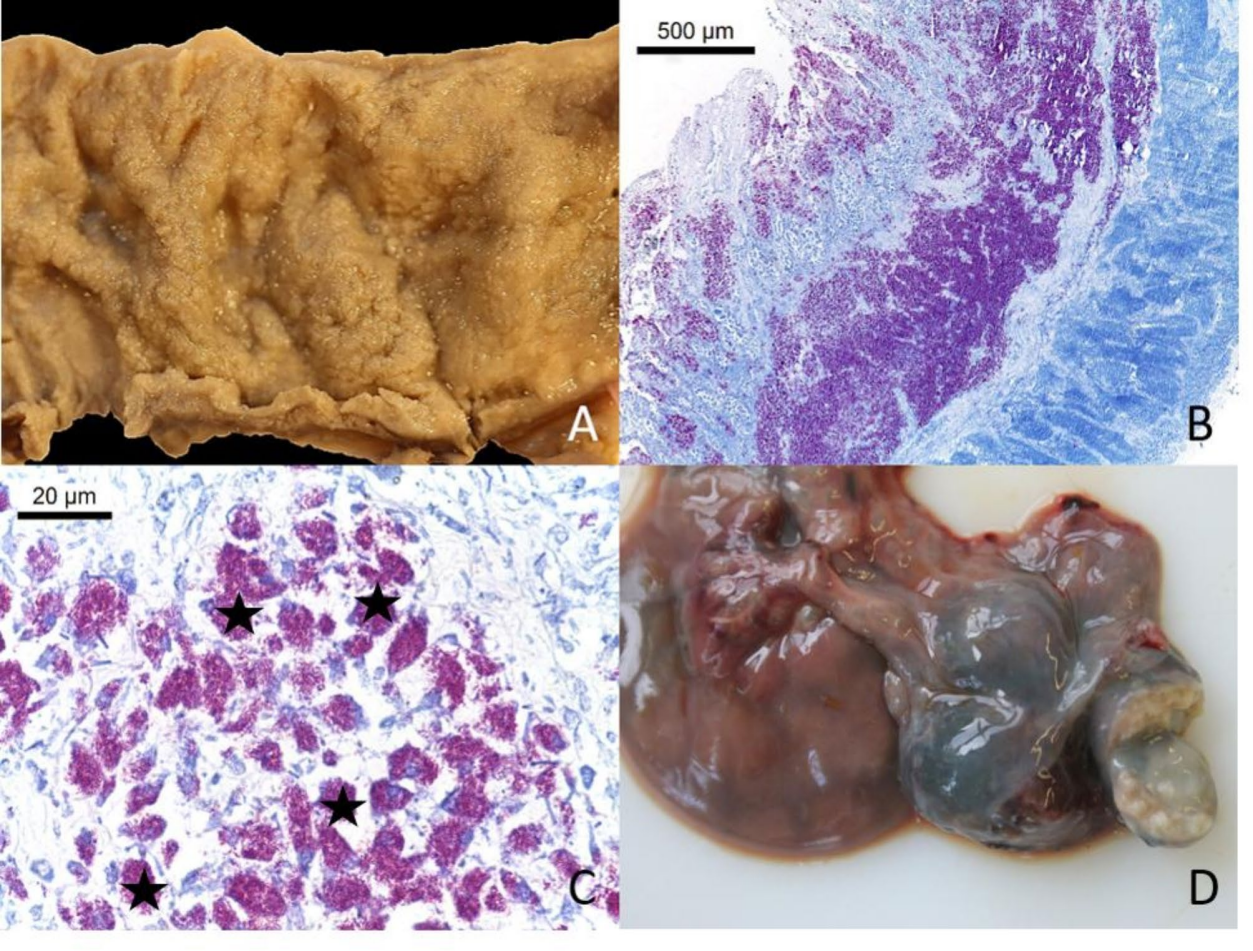
Case 1: (**A**) Small intestine with diffuse thickening of the mucosa. (**B**) severe diffuse granulomatous enteritis with abundance of acid fast bacteria in mucosa and submucosa (Ziehl-Neelsen Stain). (**C**) small intestine with intra- and extracellular acid fast bacteria within epithelioid cells (asterisks) in the Lamina propria (Ziehl-Neelsen Stain). (**D**) granulomatous lymphadenitis of the mesenteric lymph nodes

The animal was in very poor body condition with complete loss of fat or serous atrophy of adipose tissue, and the perianal region was soiled with faeces. No pleural or peritoneal effusions were observed. Severe enteritis was seen in the small intestine with watery red-brown intestinal contents. The mucosa of the small intestine starting in mid jejunum was rough and markedly thickened (Fig. 1, A). The contents of the large bowel showed no faeces formation. No macroscopically obvious lesions were observed in the mucosa of the large bowel.

Histologically the intestine revealed a severe granulomatous enteritis with epithelioid cells and abundant acid-fast, rod-shaped bacteria in the mucosa and submucosa (Fig. 1, B and C). Giant cells and mineralisation were missing. The mesenteric lymph nodes were enlarged (up to 4.0 × 3.5 × 5.0 cm) and revealed a caseous to granulomatous and necrotizing lymphadenitis (Fig. 1, D) with abundant acid-fast bacterial rods within epithelioid cells. Furthermore, the alpaca had a severe embolic-suppurative nephritis with intralesional and intravascular bacteria which were mainly extracellular and not acid-fast, and showed renal haemorrhages, interstitial oedema, fibrosis, and multifocal glomeruloscleroses. Lungs, spleen, brain, and liver were without essential pathohistological findings. The female was pregnant with one embryo (crown-rump-length: 2 cm).

#### Parasitology

Using NaNO_3_ as Flotation Medium (D (specific weight) = 1.1429–1.270) (Avantor, VWR, Germany) a low number of Coccidia-oocysts (smaller than *Eimeria macusaniensis*) could be demonstrated within the intestinal content.

#### Virology

The blood tested negative for Bluetongue virus using qPCR (virotype^®^ BTV pan/ 8 RT-PCR Kit, INDICAL BIOSCIENCE, Germany). Within the brainstem (obex) of the animal pathological prion protein was not detected (HerdCheck BSE-Scrapie Ag Test, IDEXX, The Netherlands).

#### Microbiology

Conventional cultural bacteriological examination of brain, liver, lung, small intestine and kidney on selective media revealed moderate to marked growth of *Escherichia (E.) coli var. haemolytica*. From the small intestine non-haemolytic *E. coli* were isolated as well. The intestinal content was positive for the *E. coli* intimin gene (*eae*) and negative for the Shiga toxin 1 and 2 genes (*stx1*, *stx2*) by multiplex PCR [16, 17], suggesting the presence of enteropathogenic *E. coli* (EPEC) in the intestine [18].

Genome fragments of MAP were detected in the small intestine and mesenteric lymph node by qPCR (DNA extraction: IndiMag Pathogen Kit, INDICAL BIOSCIENCE, Germany; qPCR: Adiavet ParaTB real time, Bio-X, Belgium). These samples were submitted for cultural confirmation of MAP to the National Reference Laboratory of Paratuberculosis at the FLI. Cultural isolation was performed essentially according to the protocol published by the FLI in the official manual of diagnostic procedures [19]. In brief, approx. 1 g of each tissue sample was homogenized with a stomacher and decontaminated with 7 ml of 0.9% hexadecylpyridinium chloride monohydrate solution (HPC, Sigma Aldrich, Germany). After 24 h incubation (room temperature) and centrifugation (20 min, 1,880xg, room temperature), the supernatant was discarded, and the pellet was resuspended in 1 ml 1x PBS. Of this, 200 μl each were inoculated onto three slants of Herrold’s Egg Yolk Agar with Mycobactin J and Amphotericin, Nalidixic acid and Vancomycin (HEYM, Becton Dickinson, MD, USA). Cultures were incubated for up to 16 weeks at 37 °C and regularly checked macroscopically for bacterial growth. Putative mycobacterial colonies were identified as MAP by end-point PCR targeting IS*900* [20, 21]. MAP was culturally confirmed in both tissues. The isolates were assigned to the MAP-C type by end-point PCR according to Collins et al., 2002 [22].

#### Supporting investigations

Composite faecal samples from droppings in different enclosures of the zoo (stable and runout of the alpacas, runout of Four-Horned goats, petting zoo) were examined for the presence of MAP by direct qPCR (DNA extraction: QIAamp DNA Mini Kit, Qiagen, Germany; qPCR: Adiavet ParaTB real time, Bio-X, Belgium) and culture. Genome fragments of MAP were detected in faeces from the alpaca runout and the petting zoo and MAP was culturally isolated from samples from the runout of the Four-Horned goats enclosure.

MAP had been isolated from the intestine and mesenteric lymph node of a Dwarf goat from the zoo necropsied in 2011, and genome fragments of MAP could be detected in paraffin sections of different tissues from three Four-Horned goats from the same premise submitted for necropsy to the regional veterinary diagnostic laboratory in 2017 and 2018. Furthermore, MAP was isolated from small intestine and the ileocaecal lymph node of another Four-Horned goat from the zoo that was submitted for necropsy to the regional veterinary diagnostic laboratory in 2023.

#### Molecular genetical characterization of the MAP isolates of case 1

The culturally isolated MAP strains were characterized by three molecular genotyping methods: Single nucleotide polymorphism (SNP)-based assay according to Leao et al., 2016 [23], Mycobacterial interspersed repetitive unit–variable number tandem repeat (MIRU–VNTR) analysis according to Thibault et al., 2007 [24], and short sequence repeat (SSR) analysis according to Amonsin et al., 2004 [25] based on three loci (SSR1, 8, and 9) and including some modifications as described in Fritsch et al., 2012 [26]. MIRU-VNTR results were assigned to INMV types using the INMV classification database (http://mac-inmv.tours.inra.fr/). DNA from colony material of MAP was extracted for genotyping as described in Schmidt et al., 2022 [27].

The MAP isolates from the alpaca (intestine, intestinal lymph node), the Dwarf goat, the tissues of the Four-Horned goat (intestine, ileocecal lymph node) necropsied in 2023 and from one environmental sample (faecal samples from droppings of the Four-Horned goats) were assigned to Subgroup A, Clade 1 within the MAP type C group using SNP-based assay. MIRU-VNTR- and SSR typing revealed the profiles INMV 2 and 7-4-4. This resulted in the combined genotype Clade 1 / INMV 2 / 7-4-4.

### Case 2 – alpaca from private owner

#### Anamnesis and clinical findings

The affected animal was a 2.5-year-old male Huacaya alpaca born and kept in a private husbandry in Rhineland-Palatinate, about 160 km away from case 1. The stallion was kept as one of 36 alpacas used as breeding animals. The animals were housed in a free stall barn with tightly fenced surrounding pasture.

The affected alpaca showed excessive weight loss over weeks with cachexia, weakness, heart arrhythmia, and hypothermia. In January 2022, it was transferred at the owner´s request to the Clinic for Ruminants, Faculty of Veterinary Medicine, Giessen for diagnostics and treatment.

On presentation, the 42.3 kg alpaca showed a reduced body condition. The animal was able to stand, but laid down frequently. The rectal temperature was 39 °C (normal range: 37.5–38.9 °C; [28]). The examination of the cardiovascular system revealed pale mucous membranes with a severely enhanced heart frequency of 148 beats per minute (normal range: 60–90 bpm; [28]). A systolic cardiac murmur was present in the region of the bicuspid valve. The animal had an accelerated breathing rate of 40 breaths/min with regular costoabdominal breathing (normal range: 10–30/min; [28]). No abnormalities on external clinical examination of the gastrointestinal tract (GIT) could be revealed. All peripheral lymph nodes were not palpably enlarged. Ultrasonography of the abdomen revealed no free abdominal fluid but a reduced intestinal motility. Emergency laboratory blood work displayed a mild hypochromic anaemia with PCV of 0.21 l/L (range: 0.27–0.45 l/L) and haemoglobin concentration of 5.5 mmol/L (range: 6.8–12 mmol/L) [29] with severe leucocytosis (31.4 × 10^9^/L; range: 8–22 × 10^9^/L) and neutrophilia (90.7%; range: <30%) [30]. Differential cell counts displayed 90.7% neutrophils, 6.9% lymphocytes, 0.5% monocytes, 1.8% eosinophils, and 0.1% basophils, respectively. Analysis of the electrolytes showed a mild hyponatremia with 142.7 mmol/L (range: 145–158 mmol/L) but severe hypocalcaemia with 0.84 mmol/L (range: 1.1–1.4 mmol/L) respectively. Creatinine was markedly reduced to 57.6 μmol/L (range: 97–194 μmol/L) and hyperglycaemia was present with 21 mmol/L (range: 5.-12.4 mmol/L) [29, 31].

The animal`s condition worsened rapidly despite fluid therapy and the stallion died during the first night of hospitalization. The carcass was submitted to the Institute of Veterinary Pathology of the Justus-Liebig-University of Giessen for necropsy.

#### Pathology

**Fig. 2:**
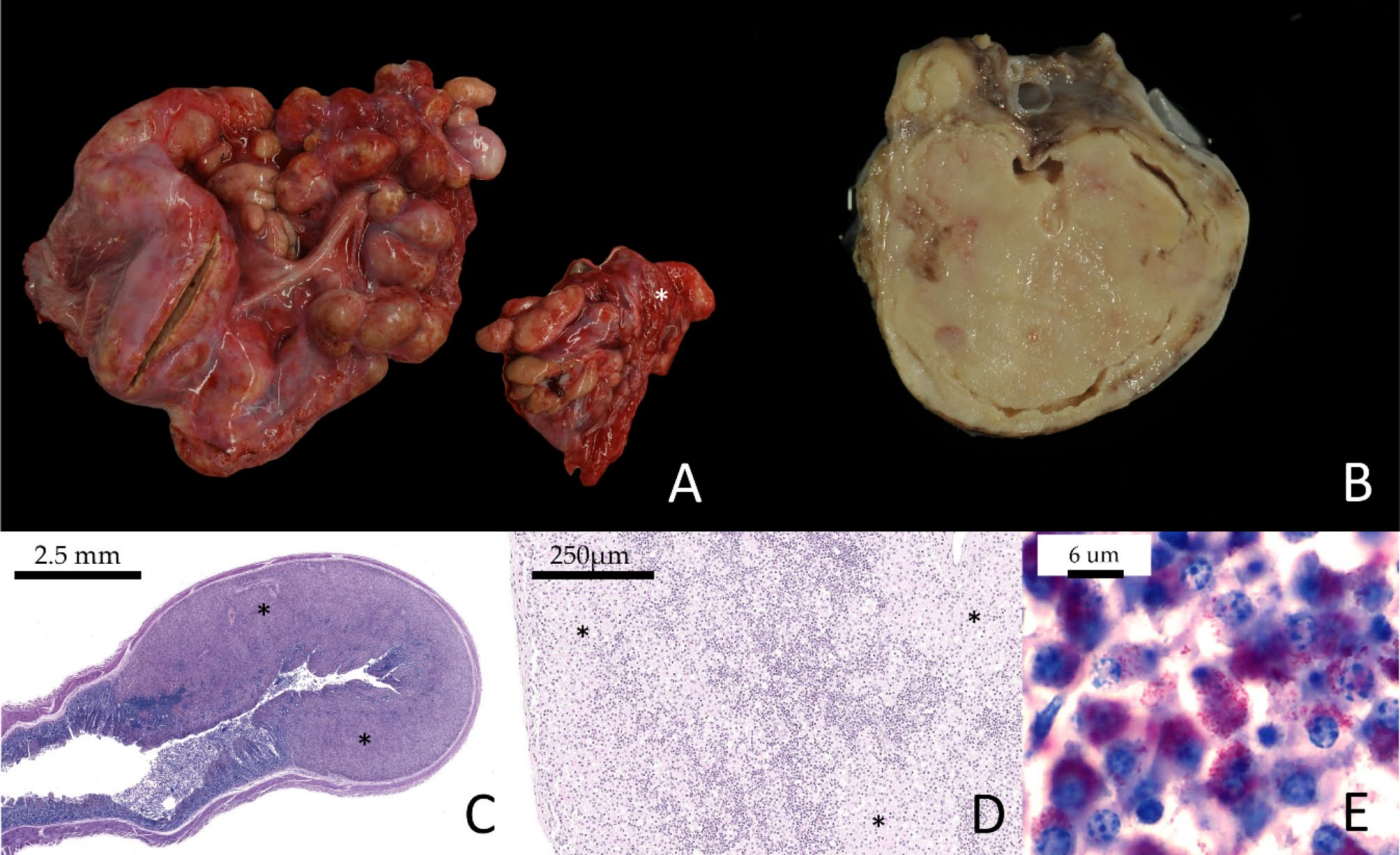
Case 2: (**A**) Gross depiction of fresh mesentery with absence of fat (cachexia), and enlargement of multiple mesenteric lymph nodes (asterisk denotes pancreas). (**B**) Formalin-fixed mesenteric lymph node displaying granulomatous lymphadenitis with a diffuse yellowish and friable cut section. (**C**) Ziehl-Neelsen-stained subgross depiction of diffuse mucosal and nodular submucosal infiltration within the small intestine (asterisks). (**D**) Hematoxylin-Eosin-stained mandibular lymph node with sheet-like to nearly diffuse infiltration of the cortex by epithelioid cells (asterisks). (**E**) Ziehl-Neelsen-stained lymph node as in D), displaying epithelioid cells infiltrating between pre-existent lymphoid tissue on higher magnification, with abundant intracytoplasmic acid-fast material

The animal was cachectic and had moderate amounts of clear pleural, pericardial, and abdominal effusions. Notably, the mandibular, parotid, mediastinal, aortic, and mesenteric lymph nodes were severely enlarged, with individual mesenteric nodes measuring up to approx. 20 × 10 × 10 cm (Fig. 2, A). All lymph nodes were yellowish and friable on cut section (Fig. 2, B). The mucosa of the distal small intestine showed multiple raised nodules up to 0.8 × 0.8 × 0.5 cm in size, with an uneven yellowish colour and a soft consistency on cut section. The small intestine contained yellow to green fluid and the intestinal serosal surfaces were multifocally reddened. In contrast, the rectum contained well-formed faeces. The third gastric component (C3) contained semi-moist, well masticated contents, and displayed mild multifocal erosions. Impression smears of mesenteric lymph nodes revealed numerous acid-fast rod-shaped bacteria. Histopathology confirmed severe chronic granulomatous lymphadenitis and enteritis with numerous, mainly intrahistiocytic acid-fast material (Fig. 2, E). Epithelioid cells obscured lymph node architecture by sheet-like to nearly diffuse infiltration into sinus, cortex and medulla (Fig. 2, D). The intestinal mucosa was diffusely infiltrated, while the submucosa was multifocally and severely infiltrated by numerous epithelioid cells, rare lymphocytes, and plasma cells, often forming submucosal nodules corresponding to grossly visible nodular intestinal lesions (Fig. 2, C). Similar to case 1, neither the lung, spleen, nor brain were affected by granulomatous inflammation. The spleen displayed mild follicular hyperplasia. Intrahistiocytic acid-fast material was rarely found in small, multifocal, randomly distributed granulomas, as well as in lymphatics and periportal areas of the liver. Necrosis in histologically examined lymph nodes was rare, and multinucleated giant cells and mineralization were absent.

#### Microbiology

The presence of mycobacteria was shown in one mesenteric lymph node after DNA extraction using an in-house method by conventional PCR targeting the 16 S rRNA encoding gene using primers TPU1for [32] and R259rev [33] by the Institute for Hygiene and Infectious Diseases of Animals, Justus Liebig University Giessen. Two samples of the small intestine as well as one mesenteric lymph node were submitted for cultural and molecular biological identification of the mycobacteria to the National Reference Laboratory of Bovine Tuberculosis at the FLI. Approx. 25 mg tissue of each sample were used to extract DNA (DNeasy Blood and Tissue Kit, Qiagen, Germany). The DNA preparations tested negative for the presence of DNA specific for *Mycobacterium tuberculosis* complex using qPCRs according to the official manual of diagnostic procedures published by the FLI [34], but positive for MAP (DNA extraction: QIAamp DNA Mini Kit, Qiagen, Germany; qPCR: Adiavet ParaTB real time, Bio-X, Belgium).

MAP was culturally isolated from all three samples essentially as described above (see case 1). Genome fragments of MAP were detected by qPCR (see above) in formalin-fixed paraffin-embedded sections of the mandibular lymph node, lung, liver and colon.

#### Supporting investigations

Faecal samples from the mother of the diseased alpaca stallion and from her recent cria, as well as composite faecal samples from droppings in the enclosures of female alpacas, male alpacas and mouflon sheep kept at the same premise were tested by qPCR and bacterial culture for MAP as described above (see case 1). Genome fragments of MAP were detected in one faecal sample of the mother and in the composite faecal sample from the enclosure of the female alpacas. Cultural isolation of MAP was not successful from any of the samples.

#### Molecular genetical characterization of the MAP isolates of case 2

Characterization of MAP isolates was done as described for case 1. The MAP isolates from small intestine and mesenteric lymph node of the male alpaca were assigned to Subgroup A, Clade 8 within the MAP type C group, using the SNP-based assay. MIRU-VNTR- and SSR typing revealed the profiles INMV 1 and 7-4-4, resulting in the combined genotype Clade 8 / INMV 1 / 7-4-4.

## Discussion and conclusions

This report provides insights into the occurrence and manifestation of paratuberculosis in alpacas in Germany. To date, paratuberculosis in German SAC has, to the best of our knowledge, only been reported once in a clinically affected 11-year-old male alpaca from another zoological garden [15]. The two cases of this report underline that MAP infection is present in alpacas in Germany not only in zoos but also in private holdings.

Clinical diagnosis of the disease is hampered by the rather non-specific symptoms like weakness and progressive weight loss. Weight loss in SAC is often obscured by their fleece [8], and the disease may therefore be easily missed by owners and veterinarians [12]. Diarrhoea is not a consistent clinical feature, as is shown in case 2 of this report. This confirms literature data in which diarrhoea was only observed in up to 50% of MAP positive SACs [8, 35]. Severe clinical courses have been observed in llamas and alpacas as early as 12–18 months of age [8, 35, 36]. On necropsy, paratuberculosis in SAC exhibits key features consistent with findings in domestic or wild ruminants, notably granulomatous lymphadenitis and enteritis. The distribution of lesions, primarily affecting the distal small intestine, colon and mesenteric lymph nodes, resembles bovine paratuberculosis cases [37]. Caseating necrosis of mesenteric lymph nodes, which is more typical for ovine, caprine and cervid MAP infections, was detected in both alpaca cases, although mineralization was absent. According to the literature, enlargement of mesenteric (including the ileocaecal) lymph nodes is most consistently observed in SAC, whereas the intestinal mucosa may be variably thickened or grossly unaffected [15, 35, 36, 38]. Thickening of the intestinal mucosa was particularly obvious in the pluribacillary paratuberculosis of case 1 of this report (Fig. 1A, B). Extraintestinal lesions of MAP infection are rarely reported and are often only detected histologically [36, 37]. In this context, the mandibular lymph nodes of case 2 were severely enlarged and showed severe granulomatous lymphadenitis. In formalin-fixed paraffin-embedded sections of this lymph node as well as in the lung and liver, MAP DNA was detected by qPCR, suggesting a causal connection between swelling of the mandibular lymph node and MAP infection. Histologically, the spleen revealed mild follicular hyperplasia, and rare multifocal granulomas containing acid-fast material were present in the liver, whereas the lung showed no inflammation. The detection of MAP DNA in the mandibular lymph node and liver might be due to direct colonization after oral ingestion, as has been suggested for tonsils, retropharyngeal and superficial cervical lymph nodes, hepatic lymph nodes and liver in goats [39]. Dissemination to extraintestinal tissues is another likely explanation, as was observed in experimentally infected goats with confluent to diffuse granulomatous intestinal lesions [39], naturally infected goats [40] and sheep [41].

According to the literature, gastrointestinal diseases are among the most common disorders in SAC and are often the immediate cause of death. *E. coli* and clostridial infections predominate among the infectious causes of intestinal inflammation [42]. It is assumed that in case 1, *E. coli* septicaemia has developed in the final stage of paratuberculosis as a result of the breakdown of the intestinal barrier and the general weakening of the animal. The septicaemia might have contributed to the final deterioration of the general condition, while emaciation and intractable diarrhoea were due to MAP infection.

The primary pathological differential diagnoses of paratuberculosis in SAC include infections with other Mycobacteria. Because of the zoonotic risk, the first priority is to rule out disseminated infections with members of the *Mycobacterium tuberculosis*-complex. Tuberculosis (TB) in SAC, mainly caused by *M. bovis* or *M. microti* is gaining importance in recent years, especially in regions where TB is endemic in cattle or wildlife [43]. Symptoms in SAC are usually associated with severe lung pathology [44], however, dissemination to the mesentery and intestine has been occasionally observed [45]. On the other hand, *Mycobacterium avium* ssp. *avium* infections have been reported, in which lesions closely resembled those of paratuberculosis [46, 47].

Depending on exact organ distribution and lymph nodes affected, other differential diagnoses on gross findings can include caseous lymphadenitis by *Corynebacterium pseudotuberculosis* [48] or *Rhodococcus hoagii* [49]. In pseudotuberculosis (*Corynebacterium pseudotuberculosis-*infection) of SAC, subcutaneous and internal lymph nodes, lungs, liver, and kidneys are most commonly affected, presenting with suppurative to pyogranulomatous inflammation without acid-fast bacilli [48]. Rhodococcosis (*Rhodococcus hoagii*-infection) in camelids (including but not limited to SAC) causes serofibrinous effusions, pyogranulomatous or suppurative to necrotizing pneumonia and lymphadenitis, granulomatous enteritis and hepatitis [49]. Intralesional bacteria, which are not acid-fast, are typically observed.

Supporting investigations in the herds of origin of both alpacas of this case report elucidated possible transmission routes of MAP. With regard to case 1, the zoo has a long history of paratuberculosis: the disease has already been diagnosed in Dwarf goats from the zoological garden in 2011 and in Four-Horned goats in 2017 and 2018, and another positive Four-Horned goat that was submitted for necropsy in 2023. MAP isolates from these goats, from droppings sampled in the enclosure of the goats and from the female alpaca represented the same MAP genotype: Clade 1 / INMV 2 / 7-4-4 (SNP-based assay / MIRU-VNTR- / SSR-typing). In addition, fragments of MAP DNA were detected in droppings in the alpaca runout and in the petting zoo. MAP is mainly transmitted directly by the faecal-oral route, including via faecal contamination of the udder or pasture [2]. This is favoured by the high tenacity of MAP in the environment; the pathogen can survive in faecal material from soil for more than six months, depending on environmental conditions [50]. Transmission of MAP between different animal species grazing on the same pasture has been reported, for example between sheep and guanacos in Patagonia [14], cattle and red deer in Germany [26], and between beef cattle and deer in New Zealand [51]. In the present case, transmission between different animal species cannot be excluded, although the different species are kept currently in separate enclosures. It is no longer possible to trace whether the enclosures were restructured in the past, as this was not recorded. On the other hand, mechanical spread of MAP between the enclosures of the different species by equipment and boots during animal care, or by visitors, must be considered. Since the 26-year-old alpaca of case 1 was born in the zoo, it cannot be clarified how MAP was introduced into the premise and which species was first infected. To date, at least three species are affected: alpacas, Four-Horned goats and Dwarf goats.

Regarding case 2, cultural isolation of MAP was only possible from tissues of the male alpaca. Fragments of MAP genome could be identified by qPCR in the faeces of the mother of the case and in samples from droppings in the enclosure of the female alpacas. Bacteriological culture was not successful. Although no viable MAP was found in the samples from the farm, transmission of MAP between the alpacas by grazing on the same pasture is the most likely route of infection. A similar scenario was reported previously on a farm of 77 alpacas in the U.S.A. with three cases of clinical paratuberculosis presented within two months [38]. Positive faecal qPCR results were obtained in 12 alpacas in the absence of positive faecal culture, and MAP was detected by qPCR in environmental faecal samples obtained from two of the three pastures of the farm.

The MAP strains from the two alpacas were assigned to MAP type-C, Subgroup A based on the results of SNP-based assay and the WGS SNP-based phylogenetic tree [23]. They belonged to different phylogenetic clades (Clade 1 and Clade 8) within Subgroup A, suggesting that there were no epidemiological links between the two cases. The SSR type (7-4-4), identical in both cases, is very common in isolates from different hosts in Germany [26, 52] and Europe [53]. However, in addition to the assignment to different phylogenetic clades, the strains differed also in their MIRU-VNTR type (INMV 2 vs. INMV 1). Both profiles are predominant in European countries, Canada, and Argentina [54–56]. Comparative studies with WGS-SNP analyses have shown, that genetic markers of MIRU-VNTR and SSR typing can overestimate or underestimate the relationship between strains [55, 57]. In this study, the different INMV profiles support the result of the SNP-based assay that the two cases were caused by different MAP strains and are, thus, epidemiologically independent.

Globally, genotyping data of MAP isolates from alpacas are rare and not directly comparable with the present results, because less complex typing protocols were applied. In general, both MAP Type-C as well as MAP Type-S, the sheep-type of MAP, strains were isolated from SAC [7, 14, 38, 58]. The SSR genotype 7-4-4 (SSR1, SSR8, and SSR9) found in this study differed to SSR genotypes 12 − 5, 7 − 6, and 7 − 5 (SSR1, SSR8) found previously in an alpaca herd in the United States. Based on the SSR8 results, these genotypes can also be assigned to MAP Type-C [25, 59]. Recently, MAP isolates with MIRU-VNTR profile INMV 2 have been isolated from free ranging guanacos in Patagonia [14]. On the other hand, identical or similar MAP genotypes have been found in other animal species in Germany. The genotype of case 1 (Clade 1 / INMV 2 / 7-4-4), has been determined in cattle from Saxony (in 2008) (Möbius, unpublished data) and in a dairy goat herd in Thuringia [52], and genotype Clade 1 / INMV 2 (without SSR-typing results) in cattle in Hesse and Thuringia, Germany [60]. The genotype of case 2 (Clade 8 / INMV 1 / 7-4-4) is present in domestic and wild ruminants in the wider geographic area of this case. It was found in cattle of three herds in North Rhine-Westphalia (2004 and 2006) and from one cattle (2010) and two red deer (2023) in Rhineland-Palatinate (Möbius, unpublished data). This underlines that the two C-type strains with different genotypes belong to the MAP population circulating in different hosts and regions in Germany.

In conclusion, MAP can cause severe clinical disease in alpacas of variable age and under different husbandry conditions. This is likely due to cross-species infection with domestic or zoo ruminants. Therefore, paratuberculosis should be considered for differential diagnosis in alpacas with emaciation and poor general condition.

## Data Availability

The data generated and analysed during the current study are available from the corresponding author on reasonable request.

## Abbreviations

(q)PCR: (quantitative) Polymerase Chain Reaction
DNA: Deoxyribonucleic acid
FLI: Friedrich-Loeffler-Institut
HEYM: Herrold´s Egg Yolk Medium
HPC: Hexadecylpyridinium chloride
IS900: Insertion sequence 900
MAP: Mycobacterium avium subsp. paratuberculosis
MIRU–VNTR: Mycobacterial interspersed repetitive unit–variable number tandem repeat analysis
PBS: Phosphate buffered saline
PCV: Packed cell volume
SAC: South American Camelids
SNP: Single nucleotide polymorphism
SSR: Short sequence repeat analysis
